# Body changes after cancer: female cancer patients’ perceived social support and their perspective on care

**DOI:** 10.1007/s00520-019-04729-w

**Published:** 2019-03-15

**Authors:** Heleen C. Melissant, Cornelia F. van Uden-Kraan, Birgit I. Lissenberg-Witte, Irma M. Verdonck-de Leeuw

**Affiliations:** 1 Department of Clinical, Neuro- and Developmental Psychology, Faculty of Behavioral and Movement Sciences, Amsterdam Public Health Research Institute, Vrije Universiteit Amsterdam, van der Boechorststraat 7, Amsterdam, Netherlands; 2 Cancer Center Amsterdam (CCA), Amsterdam UMC, Vrije Universiteit Amsterdam, de Boelelaan 1118, Amsterdam, Netherlands; 3 Amsterdam UMC, Department of Epidemiology and Biostatistics, Vrije Universiteit Amsterdam, de Boelelaan 1089a, Amsterdam, Netherlands; 4 Amsterdam UMC, Department of Otolaryngology-Head and Neck Surgery, Amsterdam Public Health Research Institute, Cancer Center Amsterdam, Vrije Universiteit Amsterdam, P.O. Box 7057, de Boelelaan 1117, Amsterdam, 1007 MB Netherlands

**Keywords:** Supportive care, Body image, Appearance, Cancer patients, Social support

## Abstract

**Purpose:**

The aim of this study was to investigate among female cancer patients their perceived social support from health care professionals (HCPs), family and friends, and public media, and their perspective on care concerning body changes.

**Methods:**

A study-specific questionnaire was completed by 235 female cancer patients. Descriptive statistics were used to describe social support and perspective on care. Logistic regression analyses were used to investigate the associations between social support and sociodemographic and clinical factors, psychosocial impact, and importance of appearance.

**Results:**

More than half of the patients received sufficient support from HCPs (54%) and family and friends (55%), and a third from the media (32%). Higher educated patients and those who found appearance not important during illness perceived lower support from HCPs. Patients without a partner, and those with a surgical treatment only, perceived lower support from family and friends. Patients who were older, higher educated, without a partner, and those who found appearance not important during illness perceived lower support from the media. In total, 15–50% of the patients received sufficient care for different domains of body changes. Patients expressed the highest need for psychological support (28%) and nutrition (28%).

**Conclusions:**

Half of the female cancer patients reported to receive sufficient social support concerning body changes after cancer. Perceived support depended on age, education, relationship status, and treatment modality. The need for more care was moderate.

**Electronic supplementary material:**

The online version of this article (10.1007/s00520-019-04729-w) contains supplementary material, which is available to authorized users.

## Introduction

Cancer and its treatment can lead to substantial body changes, such as hair loss [1], weight changes [2, 3], scars, or facial disfigurements [4]. These changes can affect the body image of cancer patients and may lead to substantial psychological distress [5, 6], feelings of embarrassment, and a negative self-esteem [7, 8]. This makes body changes and related body-image problems an important aspect of quality of life (QOL) in cancer patients [9]. Social support with respect to body changes includes listening and empathizing with the cancer patient. Supporting cancer patients in the challenges they have to deal with is important, because it can reduce distress [10, 11]. More specifically, acknowledgment of body changes can facilitate adjustment of cancer patients to their new appearance [12, 13]. Social support can be offered by various sources. First, health care professionals (HCPs) play an important role in offering guidance on body changes. However, HCPs are often hesitant to bring up the subject due to a lack of training [14] or because they perceive appearance as irrelevant or a luxury problem [15, 16]. Second, support from family and friends is essential. There is evidence that this type of support may be critical in terms of QOL outcomes and mortality in general [17]. In terms of body changes, feeling supported can be challenging, because cancer patients are sometimes (unintentionally) stigmatized by means of staring, avoidance, or by making specific statements about the way the patient looks [18]. On the other hand, it may occur that cancer patients avoid social situations in order not to be confronted with (undesirable) attention [19], making it also difficult to receive support. Third, support from the public media is a key factor in creating awareness about body changes after cancer and its impact on quality of life [20].

In addition, it is meaningful to investigate who is feeling supported on body changes from the abovementioned sources. In relation to adjustment to cancer, younger cancer patients seem to benefit more from support from the media and government than older patients [21].

In some cases, social support is not sufficient to adjust to body changes after cancer, and supportive care interventions are needed [8, 22–26]. Yet, these needs are not always met [27]. Several organizations offer practical information on, e.g., cosmetic camouflage, color and styling, prostheses, lingerie, swimwear, and wigs [28]. Also, nutrition and exercise interventions [29, 30] can be offered to patients who want to address body changes associated with weight changes. In addition, psychological support can be helpful in case of distress [31], such as avoidance of social situations due to body changes.

To improve uptake of supportive care concerning body changes after cancer, more information on the perceived social support and the perspective on care is needed. Therefore, the aim of this study was to investigate among female cancer patients their perceived social support from health care professionals (HCPs), family and friends, and the public media, and which subgroups of patients are at risk for not receiving sufficient social support. Also, we investigated their perspective on care for body changes.

## Methods

### Design and study population

The data in this cross-sectional study was collected via an online Dutch panel of a marketing agency, on behalf of “Look Good Feel Better,” an international non-profit public service program that helps women with cancer to manage appearance-related side effects of cancer treatment [32]. This panel consists of 100,000 persons and represents the Dutch adult population. Women aged 18 or older who indicated in their panel profile that they had cancer at that current moment and women who indicated in an extra prescreening question that they had cancer in the past were invited by email to complete a study-specific online questionnaire. The questionnaire consisted of items on the main outcomes (social support, and use of and need for interventions), items on sociodemographic factors (age, education level, and relationship status) and clinical factors (cancer diagnosis, time since diagnosis, treatment phase, treatment modality, and comorbidity), items on the psychosocial impact of body changes, and importance of appearance. In total, 321 women completed the questionnaire. For this study, all women diagnosed with cancer less than 10 years ago were eligible, for the reason that time passed since diagnosis may interfere with the recall of feelings and experiences. In total, 235 cancer patients (73%) fulfilled the inclusion criteria.

### Outcome measures

#### Social support

The primary outcome measure “perceived social support concerning body changes” was assessed with three study-specific items: “Sufficient attention and acknowledgment to the consequences of body changes and related feelings during cancer is offered” (1) “by health care professionals”; (2) “by people around you”; and (3) “in the media.” Questions were answered on a 5-point scale: “completely disagree,” “disagree,” “neither disagree nor agree,” “agree,” and “completely agree.”

#### Perspective on care

The perspective on care related to body changes was measured with two study-specific items. Patients could indicate (1) if they received sufficient help and advice at time of diagnosis and treatment and (2) if they would have liked more help and advice on the following: nutrition, exercise, taking care of hair/wigs, taking care of skin and nails, makeup, color and styling, lingerie and swimwear, prostheses, and psychological support. Questions were answered on a 5-point scale from “completely disagree” to “completely agree.” Since lingerie and swimwear and prosthesis care are primarily relevant for breast cancer patients, and hair/wigs care for patients who received chemotherapy, the perspective on these care types was explored among those subgroups.

#### Associated factors

Sociodemographic factors included age (categorized into < 50 years, 51–60 years, 61–65 years, and > 65 years), education level (lower-, secondary-, and higher education), and relationship status (having a partner versus single). Clinical factors included cancer diagnosis (lung, breast, skin, colorectal, gynecologic, head and neck, blood and lymphoma, and other), time since diagnosis (< 1 year, 1–2 years, 3–5 years, and > 5 years), treatment phase (currently undergoing treatment versus after treatment), treatment modality (surgery, chemotherapy or (chemo)radiotherapy, and surgery plus chemotherapy or (chemo)radiotherapy), and comorbidity (yes/no).

Psychosocial impact of body changes was measured with eight study-specific items on (1) feelings, (2) femininity, (3) self-esteem, (4) personal functioning, (5) professional functioning, (6) importance that body changes are not visible in personal life and (7) in professional life, and (8) avoiding contact with people because of body changes.

The importance of appearance was measured with a study-specific item: “I find it important to pay attention to my appearance during my illness.” All questions were answered on a 5-point scale from “completely disagree” to “completely agree.”

### Statistical analyses

Descriptive statistics were used to describe social support, sociodemographic and clinical factors, psychosocial impact of body changes, importance of appearance, and the perspective on care related to body changes. For the logistic regression analysis, responses regarding social support, psychosocial impact of body changes, and importance of appearance were dichotomized into disagree/neutral (completely disagree, disagree, and neither disagree nor agree) or agree (agree and completely agree). Regarding perspective on care, answers were categorized into disagree (completely disagree and disagree), neutral (neither disagree nor agree), or agree (agree and completely agree). Logistic regression analyses were performed to assess associations between social support and sociodemographic and clinical factors, as well as psychosocial impact. A forward selection procedure (*p* value for enter ≤ 0.1) was performed to investigate which combination of factors predicted perceived social support best. The explained variance was calculated with Nagelkerke *R*^2^. Analyses were performed using the IBM Statistical Package for the Social Science (SPSS) version 24 (IBM Corp., Armonk, NY).

## Results

### Study sample

The mean age of the patients in this study was 57 years (SD = 13), the largest group (40%) had secondary education, and the majority (62%) had a partner. Most patients were diagnosed with breast (40%) or skin (17%) cancer. Time since diagnosis ranged from 0 to 10 years, with a median of 3 years (interquartile range, 2 to 6 years). Patients reported that body changes following cancer treatment had several consequences on their life, in particular on feelings (70%), personal functioning (57%), and self-esteem (51%). About half of the patients found it important that body changes were not visible in their personal life (55%) and professional life (48%). Appearance during illness was deemed important (74%). In total, 1 out of 6 patients (15%) avoided contact with other people because of body changes. Characteristics of the study sample are described in Table 1.

**Table 1:** Patient characteristics (*n* = 235)

	N total group	%
Sociodemographic factors		
Mean age in years (SD)	57 (13)	
< 50 years	63	27
51–60 years	71	30
61–65 years	38	16
> 65 years	63	26
Education level		
Lower education	89	38
Secondary education	94	40
Higher education	50	21
Unknown	2	1
Relationship status		
Single	89	38
Having a partner	146	62
Clinical factors		
Cancer diagnosis		
Breast	94	40
Skin	39	17
Gynecologic	22	9
Colorectal	18	8
Head and neck	15	6
Blood and lymphoma	15	6
Lung	8	3
Other	21	9
Unknown	3	1
Median time since diagnosis in years (IQR)	3 (2 to 6)	
Treatment phase		
Currently undergoing treatment	143	61
After treatment	92	39
Treatment modality		
Surgery	67	29
CT or (C)RT	36	15
Surgery plus CT or (C)RT	99	42
Other	33	14
Comorbidity		
No	141	60
Yes	94	40
Psychosocial impact		
Feelings	164	70
Femininity	81	35
Self-esteem	120	51
Personal functioning	135	57
Professional functioning	90	38
Importance that body changes are not visible		
In personal life	130	55
In professional life	113	48
Avoiding contact with people because of body changes	34	15
Finding appearance important	174	74

*IQR*, interquartile range

### Perceived support from HCPs, family and friends, and public media

A small majority perceived to receive sufficient support with regard to their body changes and related feelings during cancer from HCPs (54%) and their family and friends (55%). One in three patients reported the feeling of support of cancer-related body changes from the public media (32%).

### Associations between social support and sociodemographic and clinical factors, psychosocial impact, and importance of appearance

Results of the univariate logistic regression analyses are presented in the Supplementary file. The forward selection procedure showed that perceived support from HCPs was significantly associated with education level and importance of appearance during illness, explaining 6% of the variance (Table 2). Higher educated patients (compared with lower educated patients) and those who found appearance not important during their illness (compared with those who did find appearance important during illness) perceived less support from HCPs concerning their changed body.

**Table 2:** Results of the logistic regression analyses with forward selection procedure regarding factors associated with social support from HCPs, family and friends, and public media

	Social supporters					
	Health care professionals (*n* = 235)		Family and friends (*n* = 235)		Public media (*n* = 235)	
	OR (95% CI)	Sig.	OR (95% CI)	Sig.	OR (95% CI)	Sig.
Age (years)						0.040
< 50					1	
51–60					0.66 (0.30 to 1.43)	
61–65					0.27 (0.09 to 0.80)	
> 65					1.14 (0.52 to 2.52)	
Education level^a^		0.071				0.072
Lower education	1				1	
Secondary education	0.98 (0.54 to 1.78)				0.83 (0.43 to 1.61)	
Higher education	0.46 (0.23 to 0.95)				0.38 (0.16 to 0.88)	
Relationship status				0.092		0.009
Single			1		1	
Having a partner			1.67 (0.92 to 3.03)		2.35 (1.24 to 4.45)	
Treatment modality^b^				0.002		
Surgery			1			
CT or (C)RT			3.16 (1.35 to 7.41)			
Surgery plus CT or (C)RT			2.94 (1.54 to 5.63)			
Importance of appearance		0.015				0.008
Finding appearance not important	1				1	
Finding appearance important	2.11 (1.15 to 3.85)				2.77 (1.30 to 5.90)	
Explained variance (Nagelkerke’s *R*^2^)	0.06		0.10		0.16	

^a^Patients with an unknown education level were excluded from this analysis (*N* = 2)

^b^Patients with treatment modality “other” were excluded from this analysis (*N* = 33)

Perceived support from family and friends was significantly associated with relationship status and treatment modality, explaining 10% of the variance. Patients without a partner and who received a surgical treatment only (versus a treatment including chemotherapy or (chemo)radiation) perceived less support from family and friends.

Perceived support from the public media was significantly associated with age, education level, relationship status, and importance of appearance, explaining 16% of the variance. Patients 61–65 years old (compared with patients aged < 50 years old), higher educated patients, patients without a partner, and those who found appearance not important during their illness perceived less support from the public media.

### Perspective on care

Overall, 15–50% of the patients indicated to receive sufficient care on different domains of body changes during treatment (Table 3). Most often, patients received sufficient care on exercise (50%), nutrition (44%), psychological support (42%), and taking care of the skin (41%). Less often, taking care of nails (20%), makeup (19%), and color and styling (15%) was mentioned. In total, 9–28% of the patients needed more care, especially on psychological support (28%), nutrition (28%), taking care of skin (26%), and exercise (25%), and to a lesser extent on makeup (14%) and color and styling (9%). Among breast cancer patients, 37% reported to receive sufficient care on lingerie and swimwear and 55% of prostheses, and respectively 19% and 23% needed more care on these topics. Of the patients treated with chemotherapy, 67% reported to receive sufficient care on taking care of hair/wigs, and 22% needed more care on this topic.

**Table 3:** Female cancer patients’ perspective on care regarding body changes

	Sufficient care			Need for more care		
	Disagree, *n* (%)	Neutral, *n* (%)	Agree, *n* (%)	Disagree, *n* (%)	Neutral, *n* (%)	Agree, *n* (%)
All patients (*n* = 235)						
Nutrition	65 (28)	67 (29)	103 (44)	101 (43)	69 (29)	65 (28)
Exercise	54 (23)	64 (27)	117 (50)	101 (43)	75 (32)	59 (25)
Taking care of skin	66 (28)	72 (31)	97 (41)	99 (42)	76 (32)	60 (26)
Taking care of nails	105 (45)	83 (35)	47 (20)	115 (49)	77 (33)	43 (18)
Makeup	92 (39)	99 (42)	44 (19)	117 (50)	85 (36)	33 (14)
Color and styling	107 (46)	92 (39)	36 (15)	124 (53)	90 (38)	21 (9)
Psychological support	68 (29)	68 (29)	99 (42)	95 (40)	75 (32)	65 (28)
Breast cancer patients (*n* = 94)						
Lingerie and swimwear	31 (33)	28 (30)	35 (37)	36 (38)	40 (43)	18 (19)
Prostheses	19 (20)	23 (25)	52 (55)	36 (38)	36 (38)	22 (23)
Patients treated with chemotherapy (*n* = 87)						
Hair/wigs	12 (14)	17 (20)	58 (67)	39 (45)	29 (33)	19 (22)

## Discussion

Half of the female cancer patients in this study received sufficient social support concerning body changes after cancer, mostly from HCPs and family and friends. A third reported sufficient attention from the public media on this matter. These results indicate that there are also many cancer patients who did not receive social support. Our study showed that especially patients who were older, higher educated, without a partner, and treated with surgery only were at risk of perceiving lower support. In this study, up to half of the female cancer patients indicated to receive sufficient care with regard to body changes during treatment, and up to a third needed more care. Of all breast cancer patients, 37% received sufficient care regarding lingerie and swimwear and 55% on prostheses, and the majority of patients after chemotherapy received sufficient care for their hair/wigs (67%).

The results of this study showed that support from HCPs can be further improved. A particular interesting finding is that patients who found appearance not important during their illness perceived less support from HCPs than patients who did find that important. This attribute can also be referred to as body image investment, the value or importance one places on appearance and physical attributes [33]. It may be that body changes are discussed more adequately in consults with the HCP if patients have a higher body image investment. Whether the “looks” of a patient (having invested in appearance with, for example, a wig or makeup) triggers a conversation about body changes in a HCP, or whether patients with a high body investment bring up this topic themselves, should be investigated further.

This study also demonstrated that not all patients perceived to receive enough support from family and friends, although cancer patients primarily depend on and benefit from support from this source [21]. Especially patients who had undergone surgical treatment were at risk of perceiving lower support from family and friends, compared with patients who also received chemotherapy, radiotherapy, or both. Patients undergoing chemotherapy or radiotherapy might receive more support for their changed body because of the highly visible hair loss [1] or weight changes [34] in addition to surgical body changes. Furthermore, patients without a partner were at risk of lower perceived support from family and friends. Prior research has demonstrated that physical attractiveness is considered more important in short-term (sexual) relationships than in long-term romantic relationships [35]. Patients without a partner might have more concerns over their appearance if they would like to engage in a new relationship and might feel a lack of support for this issue. Moreover, individuals with partners have positive thoughts about their partner’s physical attractiveness (i.e., people rate their partners as more attractive than their partners would rate themselves) [36], which might be a protective factor against appearance concerns. Cancer patients without a partner might therefore need extra support from other persons close to them, such as family and friends.

Our study revealed that only 32% of female cancer patients felt supported of body changes by the public media. Media campaigns on cancer particularly focus on screening and prevention [37]. It might help cancer patients to raise awareness via public media of the consequences of cancer and its treatment on body changes. In particular, patients aged 61–65 years were at risk of lower perceived support on body changes by the media compared with patients < 50 years. The media have shown to put value on “what is beautiful is good,” where being young and attractive is idealized [38, 39]. This might explain why older women felt least supported by the media.

Overall, the percentage of explained variance (Nagelkerke’s *R*^2^) of the multivariate models was low: 6–16%. Clinical factors including treatment phase, time since diagnosis, and cancer diagnosis and psychosocial impact factors were not associated with feeling supported. It seems that besides the variables included in this study, other factors are accountable. In future research, the role of the partner and the quality of the relationship should be further investigated, because the presence of a partner seems to be of importance when appearance changes are discussed in clinical consultations [40] and patients with higher relationship quality seem to have less appearance concerns [41].

Another aim of this study was to describe female cancer patients’ perspective on care specifically addressing body changes. In comparison to the other appearance-related topics, female cancer patients reported a need for more care on nutrition (28%) and exercise (25%). Many patients also received (sufficient) care regarding these issues (44% and 50%, respectively). The results of this study are similar to other studies where high supportive care needs were reported regarding lifestyle programs, including nutrition and exercise [42]. These are essential lifestyle factors that contribute to a healthy weight. Weight gain is a frequently reported side effect of adjuvant treatment among, e.g., breast cancer patients [34], and is associated with body image concerns [43]. By referring patients to these lifestyle programs, appearance-related supportive care needs of female cancer patients might be more adequately met. It would be interesting for future studies to investigate if cancer patients can also feel supported of their body changes by other low-intensive interventions, such as through online self-management interventions in which attention is paid to body changes. For example, My Changed Body, a web-based writing activity based on self-compassion, has proven to be effective to improve body image among breast cancer survivors [44].

A strength of this study is that we investigated a broad sample of female cancer patients with different tumor types, treatment phases, and treatment modalities. However, the present study also had some limitations. The data were self-reported, which could have led to a different interpretation of the questions. For example, some patients who still visited the hospital for follow-up consults might have regarded this as being under treatment, although they had already completed their primary cancer treatment. Therefore, results should be interpreted with caution. Additionally, mainly patients with breast and skin cancers were represented in this study sample, which may limit the generalizability of the results.

## Conclusion

This study provides insight into the perceived social support on body changes among female cancer patients. The results showed that body changes due to a cancer treatment have consequences on how patients felt and on their self-esteem. Regression analyses revealed that particular patients did not feel supported in this by their HCPs, family and friends, and especially the public media. Timely support and guidance should be offered to patients who need it.

## Electronic supplementary material

ESM 1 (DOCX 23 kb)
