# Supplementary material for: Body changes after cancer: female cancer patients’ perceived social support and their perspective on care
Source: Support Care Cancer. 2019 Mar 15;27(11):4299–306. doi: 10.1007/s00520-019-04729-w (PMC6803574; doi:10.1007/s00520-019-04729-w)
Supplement: Supplementary file 1 — (DOCX 23 kb) [file 520_2019_4729_MOESM1_ESM.docx]

Body changes after cancer: female cancer patients’ perceived social support and their perspective on care

Heleen C. Melissant, Cornelia F. van Uden-Kraan, Birgit. I. Lissenberg-Witte, Irma M. Verdonck-de Leeuw

Corresponding author:

Irma M. Verdonck-de Leeuw

Department of Otolaryngology-Head and Neck Surgery

VU University Medical Center

E-mail: [im.verdonck@vumc.nl](mailto:im.verdonck@vumc.nl).

Supportive Care in Cancer

**Supplementary file.** Results of the univariate logistic regression analyses regarding factors associated with social support by HCPs, family and friends, and the public media.

|  | Social support by... | | | | | |
| --- | --- | --- | --- | --- | --- | --- |
|  | Health care professionals  (*n* = 235) | | Family and friends  (*n* = 235) | | Public media  (*n* = 235) | |
|  | OR [95% CI] | Sig. | OR [95% CI] | Sig. | OR [95% CI] | Sig. |
| Age (years) |  | 0.32 |  | 0.53 |  | 0.048 |
| <50 | 1 |  | 1 |  | 1 |  |
| 51-60 | 1.00 [0.51 to 1.96] |  | 0.78 [0.39 to 1.54] |  | 0.78 [0.38 to 1.62] |  |
| 61-65 | 0.87 [0.39 to 1.95] |  | 0.89 [0.40 to 1.99] |  | 0.35 [0.13 to 0.96] |  |
| >65 | 1.69 [0.83 to 3.43] |  | 1.30 [0.64 to 2.65] |  | 1.40 [0.68 to 2.87] |  |
| Education level |  | 0.097 |  | 0.48 |  | 0.22 |
| Lower education | 1 |  | 1 |  | 1 |  |
| Secondary education | 1.00 [0.56 to 1.81] |  | 1.15 [0.64 to 2.07] |  | 0.92 [0.50 to 1.69] |  |
| Higher education | 0.50 [0.25 to 1.01] |  | 0.75 [0.38 to 1.51] |  | 0.50 [0.23 to 1.12] |  |
| Relationship status |  | 0.20 |  | 0.30 |  | 0.012 |
| Single | 1 |  | 1 |  | 1 |  |
| Having a partner | 1.41 [0.83 to 2.39] |  | 1.33 [0.78 to 2.25] |  | 2.15 [1.18 to 3.91] |  |
| Cancer diagnosis |  | 0.34 |  | 0.077 |  | 0.88 |
| Breast | 1 |  | 1 |  | 1 |  |
| Skin | 0.54 [0.25 to 1.15] |  | 0.48 [0.23 to 1.02] |  | 0.86 [0.39 to 1.92] |  |
| Colorectal | 0.49 [0.18 to 1.38] |  | 0.24 [0.08 to 0.73] |  | 0.39 [0.10 to 1.44] |  |
| Gynecologic | 1.35 [0.52 to 3.53] |  | 0.62 [0.24 to 1.58] |  | 1.11 [0.42 to 2.91] |  |
| Head & neck | 1.16 [0.38 to 3.52] |  | 1.24 [0.39 to 3.93] |  | 0.97 [0.31 to 3.08] |  |
| Blood & lymphoma | 1.16 [0.38 to 3.52] |  | 0.93 [0.31 to 2.84] |  | 1.29 [0.42 to 3.95] |  |
| Lung | 2.32 [0.44 to 12.1] |  | 4.35 [0.51 to 36.8] |  | 0.65 [0.12 to 3.38] |  |
| Other | 0.58 [0.22 to 1.51] |  | 0.56 [0.22 to 1.46] |  | 0.78 [0.27 to 2.19] |  |
| Time since diagnosis |  | 0.18 |  | 0.84 |  | 0.72 |
| <1 year | 1 |  | 1 |  | 1 |  |
| 1-2 years | 0.48 [0.18 to 1.26] |  | 0.71 [0.28 to 1.81] |  | 0.60 [0.23 to 1.53] |  |
| 3-5 years | 0.53 [0.21 to 1.39] |  | 0.66 [0.27 to 1.65] |  | 0.74 [0.30 to 1.86] |  |
| >5 years | 0.36 [0.14 to 0.92] |  | 0.69 [0.28 to 1.69] |  | 0.65 [0.26 to 1.60] |  |
| Currently undergoing treatment |  | 0.53 |  | 0.23 |  | 0.62 |
| Yes | 1 |  | 1 |  | 1 |  |
| No | 0.85 [0.50 to 1.43] |  | 0.72 [0.43 to 1.23] |  | 0.87 [0.49 to 1.52] |  |
|  |  |  |  |  |  |  |
| Treatment modality |  | 0.15 |  | 0.003 |  | 0.60 |
| Surgery | 1 |  | 1 |  | 1 |  |
| CT or (C)RT | 1.94 [0.85 to 4.42] |  | 2.97 [1.28 to 6.89] |  | 1.33 [0.56 to 3.13] |  |
| Surgery plus CT or (C)RT | 1.75 [0.93 to 3.26] |  | 2.82 [1.48 to 5.34] |  | 1.40 [0.72 to 2.72] |  |
| Comorbidity |  | 0.52 |  | 0.49 |  | 0.50 |
| No | 1 |  | 1 |  | 1 |  |
| Yes | 0.84 [0.50 to 1.42] |  | 0.83 [0.49 to 1.40] |  | 0.82 [0.47 to 1.44] |  |
| Psychosocial impact ^a^ |  |  |  |  |  |  |
| Feelings | 1.01 [0.62 to 1.91] | 0.76 | 1.00 [0.57 to 1.75] | 0.99 | 0.83 [0.46 to 1.50] | 0.54 |
| Femininity | 0.97 [0.56 to 1.66] | 0.91 | 1.12 [0.65 to 1.93] | 0.67 | 0.90 [0.51 to 1.61] | 0.73 |
| Self-esteem | 0.98 [0.59 to 1.63] | 0.93 | 1.08 [0.65 to 1.81] | 0.77 | 0.74 [0.43 to 1.29] | 0.29 |
| Personal functioning | 1.04 [0.62 to 1.75] | 0.87 | 0.99 [0.59 to 1.67] | 0.98 | 0.75 [0.43 to 1.30] | 0.30 |
| Professional functioning | 0.85 [0.50 to 1.44] | 0.54 | 1.04 [0.62 to 1.77] | 0.87 | 0.91 [0.52 to 1.61] | 0.75 |
| Importance that body changes are not visible: ^a^ |  |  |  |  |  |  |
| In personal life | 1.44 [0.86 to 2.42] | 0.16 | 1.20 [0.72 to 2.01] | 0.49 | 1.08 [0.62 to 1.87] | 0.79 |
| In professional life | 1.26 [0.76 to 2.11] | 0.37 | 1.23 [0.73 to 2.05] | 0.44 | 1.12 [0.65 to 1.94] | 0.69 |
| Avoiding contact with people because of body changes |  | 0.41 |  | 0.62 |  | 0.43 |
| No | 1 |  | 1 |  | 1 |  |
| Yes | 0.74 [0.36 to 1.52] |  | 1.21 [0.58 to 2.52] |  | 1.36 [0.64 to 2.88] |  |
| Importance of appearance |  | 0.023 |  | 0.10 |  | 0.007 |
| No | 1 |  | 1 |  | 1 |  |
| Yes | 2.00 [1.10 to 3.60] |  | 1.63 [0.91 to 2.93] |  | 2.71 [1.32 to 5.58] |  |

^a^ The patient group with no reported psychosocial impact / no importance that body changes are not visible, was selected as reference group.
